# Caffeine is negatively associated with depression in patients aged 20 and older

**DOI:** 10.3389/fpsyt.2022.1037579

**Published:** 2022-12-01

**Authors:** Jing Bao, Peile Li, Yang Guo, Yanxu Zheng, Michael Smolinski, Jinshen He

**Affiliations:** ^1^Department of Orthopaedic Surgery, Third Xiangya Hospital of Central South University, Changsha, Hunan, China; ^2^Department of Orthopaedic Surgery, University of Pittsburgh, Pittsburgh, PA, United States

**Keywords:** caffeine intake, depression, NHANES, PHQ-9, cross-sectional study

## Abstract

**Introduction:**

Previous studies have observed the association between caffeine intake and depression, but few have considered the potential threshold effect of this issue. Therefore, the study aimed to examine the association between caffeine consumption and depression in patients aged 20 years or older using curve fitting analysis.

**Methods:**

The population was 3,263 patients from the 2017 to 2018 National Health and Nutrition Examination Survey (NHANES) with reliable answers to questions of caffeine intake and depression. Participants’ depression levels were assessed using the 9-item Patient Health Questionnaire (PHQ-9) depression scale and the caffeine consumption were investigated in a private room of NHANES. The confounding variables of this study included level of education, monthly sleepiness, age, marital status, race, cigarette smoking, sex and recreational activities.

**Results:**

In linear regression analysis, patients with a higher PHQ-9 score tend to have less caffeine intake. A similar conclusion was drawn in logistic regression model using PHQ-9 ≥ 10 as a cut-off score for depression. But when caffeine intake exceeded 90 mg, there was no significant association between caffeine intake and depression based on the curve fitting analysis.

**Discussion:**

These results suggest that people can consume some caffeine to reduce depression. But further study is needed to examine the precise causal relationship between these factors.

## Introduction

Depression, which has become one of the most common mental disorders, is experienced by a significant number of people globally and considered a primary care disease (1). It was found that one-third of adults in the United States will be affected by depression during their lives (1). According to the World Health Organization, nearly 350 million people suffer from depression globally (2). Based on data from the US. National Health and Nutrition Examination Survey (NHANES) study in 2005–2008, the prevalence of depressive symptoms and severe depression was 22 and 0.6%

(a point prevalence), respectively (3). In recent years, some studies have focused on the association between caffeine intake and depressive symptoms (4–6). When focusing on diet-related factors, it was found that coffee was one of the most popular drinks worldwide. Several epidemiological studies have found that caffeine use has a protective effect against cognitive impairment/decline (7). Another study pointed out that combined caffeine and glucose could increase the efficiency of the attentional system (8). It is concluded that caffeine has an effect on depression.

Previous studies on this issue have not reached a consensus. An animal study had researched the causal association between caffeine consumption and depressive-like mood alterations, and found the caffeine-induced A2AR blockade has therapeutically effect on depression (4). A study based on data from NHANES from 2005 to 2006 found an inverse association between caffeine intake and depressive symptoms in US adults (6). This study was supported by a similar study conducted on 9,576 participants in Korea (5). Another case-control study conducted in 2021 found a positive association between caffeine and depression in children (9). However, the samples used in these former studies were not nationally representative (9), or too old (6) to represent current conditions. Most importantly, the potential threshold effect of the association was of less concern in the former study (6).

Thus, the aim of this study was to examine the association between caffeine consumption and depression using data from the NHANES 2017–2018 database and curve fitting analysis. It is hypothesized that within a certain range greater caffeine intake could protect against depression.

## Materials and methods

### Study design

The NHANES program began in the early 1960s. The survey examines a nationally representative sample of about 5,000 people each year. The NHANES interview includes demographic, socioeconomic, dietary, and health-related questions. The examination component consists of medical, dental, and physiological measurements, as well as laboratory tests administered by trained medical personnel.

### Study participants

**FIGURE 1:**
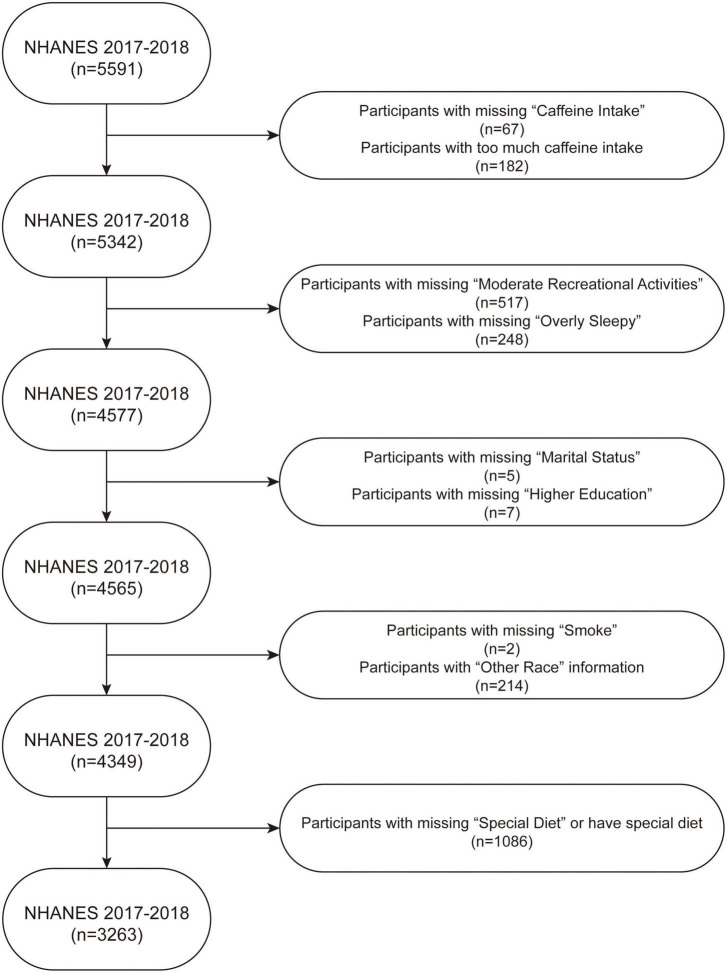
Flow chart of sample selection from the NHANES 2017–2018.

The study participants were based on the NHANES database 2017–2018 (Figure 1). Participants with missing caffeine intake information (*n* = 67), too much caffeine intake (>500 mg) (*n* = 182), missing moderate recreational activities information (*n* = 517), missing monthly sleepiness information (*n* = 248), missing marital status information (*n* = 5), missing higher education information (*n* = 7), missing smoking status information (*n* = 2), participants whose race information is “other race” (*n* = 214), and participants with special diets or missing answers of this issue (*n* = 1086) were excluded from the study. After exclusions a total of 3,263 participants were included in the analysis. Approval for this study was obtained from the ethics review board of the NHANES 2017–2018 (*n* = 5591) National Center for Health Statistics and written consent was obtained from every participant.

### Variables

The exposure variable of the study is participants with caffeine intake. In-person interviews were conducted in a private room in NHANES to obtain detailed dietary intake information to estimate the types and amounts of food and beverage (including all types of water) consumed during the 24-h period prior to the interview. NHANES calculated the energy and 64 nutrients (including caffeine) of each food using the USDA’s Food and Nutrient Database for Dietary Studies, and estimated patients’ caffeine intake. After excluded those on a special diet, the present study used the data to represent the daily caffeine consumption of patients. In the linear regression model, caffeine intake was divided into three trisection parts: Q1 (≤39 mg), Q2 (≥39 mg and ≤144 mg), and Q3 (≥144 mg and <500 mg) to calculate the P for trend. The detailed data can be accessed in the Total Nutrient Intakes, First Day of Dietary Interview.

The outcome variable was depression. NHANES assessed the levels of depression using the 9-item Patient Health Questionnaire (PHQ-9) depression scale, which consists of nine questions based on symptoms of depression. Each answer to the nine questions is scored 0 to 3, with 0 (not at all), 1 (several days), 2 (more than half the days), and 3 (nearly every day). The scores are summed to a total score between 0 and 27. In the linear regression model and curve fitting analysis, the initial scores of PHQ-9 (a continuous variable), from 0 to 27 were used. The present study used an PHQ-9 score ≥10 as a cut-off score for depression, as it has a sensitivity of 88% and a specificity of 88% (10), and conducted the logistic regression analysis. These are contained in the Mental Health—Depression-screener data of Questionnaire Data section in NHANES.

Eight confounding factors were included in this study. The categorical variables included higher education, monthly sleepiness, marital status, race, cigarette smoking, and sex. The continuous variables included age and moderate recreational activities. The race consisted of four parts, with “Latin” including those of Hispanic white and Mexican background. Data for moderate recreational activities in a week was a product of “days recreational activities” and “minutes recreational activities” in NHANES. Higher education had two answers, “yes” which included college graduates and those with college degrees or Associates of Arts degrees, while “no” included those who had not entered college. Smoking status in the past 30 days was a continuous variable of frequency of smoking per day in the past 30 days and was categorized as “no” (= 0) and “yes” (>0). Monthly sleepiness is the frequency of overly sleepiness during the past month before patients answered the question. Marital status included “current” (married, living with partners), “past” (widowed, divorced, or separated), and “never” (never married). All these variables are available in NHANES.

### Statistical analysis

The present study conducted the linear regression analysis and curve fitting analysis using the PHQ-9 score to examine the association between caffeine consumption and depression. Then the categorized depression (depression or non-depression) was used to conduct the logistic regression analysis. Subgroup analysis using linear regression analysis and curve fitting analysis were also performed. All analyses were conducted using EmpowerStats software (version 3.0) and the R Project for Statistical Computing (version 3.2.3), and *p* < 0.05 was considered statistically significant.

## Results

A total of 3,263 participants (aged 20 years or older) were included in this analysis and divided into two groups: non-depression (*n* = 3039) and depression (*n* = 244), as shown in Table 1. There were statistically significant differences in caffeine intake, marital status, monthly sleepiness, moderate recreational activities, age, education level, smoking status, and sex between the groups. Compared to patients without depression, depressive patients are more likely to be female, younger, sleepier, have been married but broken up or never married, have not received higher education, have less than moderate recreational activities, tend to smoke, and have less caffeine intake.

**TABLE 1:** The characteristics of the study participants in the NHANES 2017–2018.

	Non-depression (*n* = 3019)	Depression (*n* = 244)	*P*-Values
Caffeine intake (mg)	143.2 ± 122.1	126.3 ± 122.8	0.05
Moderate recreational activities (minutes per week)	93.0 ± 186.8	58.3 ± 128.2	0.006
Age (years)	48.1 ± 17.6	45.0 ± 17.5	0.01
Higher education* (%)	60.5	53.2	0.03
Marital status (%)			<0.0001
Current	63.4	44.0	
Past	17.9	27.5	
Never	18.7	28.5	
Monthly sleepiness (%)			<0.0001
Never (0)	13	5.1	
Rarely (1 time)	27	6.3	
Sometimes (2–4 times)	36	32.9	
Often (5–15 times)	18	34.9	
Always (16–30 times)	6	20.8	
Race (%)			0.15
Latin	16.5	17	
Non-Hispanic white	64.8	64	
African American	12.5	16	
Asian	6.2	3	
Cigarette smoking (%)	15.1	35.1	<0.0001
Male (%)	48.9	41.2	0.03

Mean ± SD for continuous variables; % for categorical variables. *“Higher Education” means whether or not patients have entered college.

Linear regression analysis was used to estimate the association between caffeine and depression and the results of which are presented in Table 2. In the non-adjusted model, patients with higher PHQ-9 score were more likely to have less caffeine intake (β: −0.002, 95% CI: −0.003 to −0.0006, *P* for trend: 0.002). The same conclusion was drawn according to the minimally adjusted model (β: −0.002, 95% CI: −0.003 to −0.0003, *P* for trend: 0.02) and fully adjusted model (β: −0.002, 95% CI: −0.003 to 0.0005, *P* for trend: 0.004). In the logistic regression model (as shown in Table 3), the results also indicated a negative association between caffeine consumption and depression (OR: 0.998, 95% CI: 0.9967 to 0.9991, *P* < 0.001).

**TABLE 2:** Association between caffeine intake and depression (linear regression model).

Exposure	Model 1 β (95% CI)	Model 2 β (95% CI)	Model 3 β (95% CI)
Caffeine	−0.002 (−0.003, −0.0006)	−0.002 (−0.003, −0.0003)	−0.002 (−0.003, −0.0005)
**Caffeine consumption**			
Q1	Reference	Reference	Reference
Q2	−0.66 (−1.05, −0.27)	−0.65 (−1.04, −0.26)	−0.67 (−1.03, −0.31)
Q3	−0.71 (−1.06, −0.36)	−0.63 (−1.00, −0.26)	−0.56 (−0.90, −0.21)
*P* for trend	0.002	0.02	0.004

Non-adjusted model (Model 1): None. Minimally adjusted model (Model 2): Age; sex; race. Fully adjusted model (Model 3): Higher education; marital status; monthly sleepiness; moderate recreational activities; race; cigarette smoking; sex and age.

**TABLE 3:** Association between caffeine intake and depression (logistic regression model).

	Model 1	Model 2	Model 3
Non-depression (Reference)	1	1	1
**Depression**			
OR (95% CI)	0.999 (0.997, 0.9996)	0.998 (0.9967, 0.9991)	0.998 (0.9967, 0.9991)
*P*	0.008	<0.001	<0.001

Non-adjusted model (Model 1): None. Minimally adjusted model (Model 2): Age; sex; race. Fully adjusted model (Model 3): Higher education; marital status; monthly sleepiness; moderate recreational activities; race; cigarette smoking; sex and age.

**FIGURE 2:**
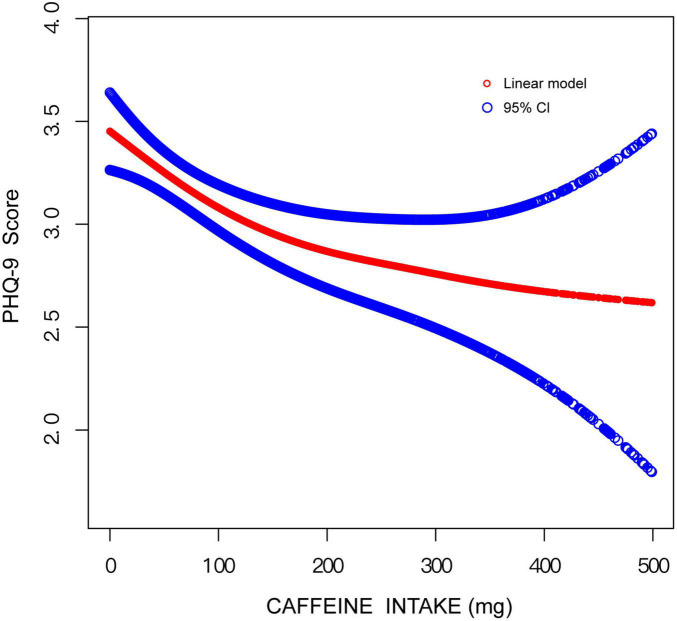
Curve fitting model on the association between caffeine intake and depression.

More detailed results were found using curve fitting analyses (Figure 2). A negative relationship between caffeine and depression was found when caffeine intake was lower than 90 mg. However, when caffeine intake was greater than 90 mg, there was not a significant association between caffeine intake and depression.

**FIGURE 3:**
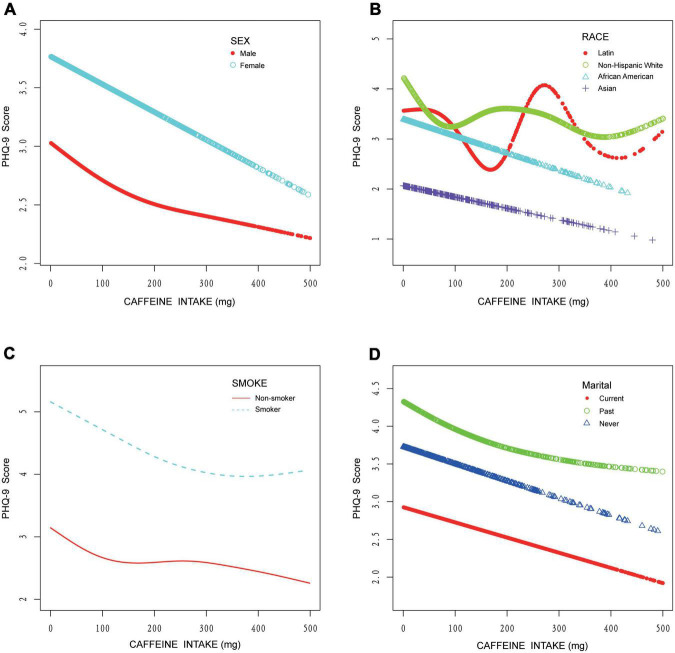
Curve fitting model on the association between caffeine intake and depression stratified by sex, race, cigarette smoking, and marital status. **(A)** Stratified by sex. **(B)** Stratified by race. **(C)** Stratified by cigarette smoking. **(D)** Stratified by marital status.

In subgroup analyses, results of multivariate regression analysis stratified by education level, smoking status, sex, and marital status are presented in Table 4. The association between caffeine intake and depression only exists in those who do not smoke (β: −0.002, 95% CI: −0.003 to −0.0004, *P*: 0.01) and the “never” group in marital status (β: −0.004, 95% CI: −0.007 to −0.0004, *P*: 0.03). The association between caffeine consumption and depression was more apparent in those who have not received higher education (β: −0.002, 95% CI: −0.004 to −0.0003, *P*: 0.03) in subjects with high-level education level and in females (β: −0.002, 95% CI: −0.004 to −0.0001, *P*: 0.04). More detailed information could be seen in Table 4. In curve fitting analysis, for three kinds of patients (smoking, female or married in the past), the negative association between caffeine and depression was significant only when caffeine is lower than respective certain doses (as showed in Figures 3A,C,D). But for Latin and non-Hispanic white, the situation is more complex (also showed in Figures 3B).

**TABLE 4:** Association between caffeine intake and depression, stratified by higher education, sex, marital status, and cigarette smoking.

Caffeine	Model 1	Model 2	Model 3
**Higher education**			
**Yes**			
β (95% CI) *P*	−0.0024 (−0.004, −0.001) 0.001	−0.0021 (−0.004, −0.0005) 0.008	−0.0015 (−0.003, −0.0001) 0.03
**No**			
β (95% CI) *P*	−0.0006 (−0.0025, 0.0013) 0.55	−0.001 (−0.0027, 0.0013) 0.50	−0.002 (−0.0043, −0.0003) 0.03
**Marital status**			
**Current**			
β (95% CI) *P*	−0.002 (−0.004, −0.001) 0.001	−0.0006 (−0.002, 0.001) 0.43	−0.0010 (−0.002, 0.0004) 0.16
**Past**			
β (95% CI) *P*	−0.0029 (−0.006, −0.0002) 0.04	−0.0025 (−0.005, 0.0004) 0.09	−0.0015 (−0.004, 0.001) 0.29
**Never**			
β (95% CI) *P*	−0.0034 (−0.0066, −0.0002) 0.04	−0.0038 (−0.0072, −0.0004) 0.03	−0.0035 (−0.0066, −0.0004) 0.03
**Sex**			
**Male**			
β (95% CI) *P*	−0.0017 (−0.003, −0.0001) 0.04	−0.0013 (−0.003, 0.0003) 0.10	−0.0015 (−0.003, 0.0000) 0.052
**Female**			
β (95% CI) *P*	−0.0017 (−0.003, 0.0001) 0.06	−0.0017 (−0.004, 0.0002) 0.08	−0.0018 (−0.004, −0.0001) 0.04
**Cigarette smoking**			
**Yes**			
β (95% CI) *P*	−0.0027 (−0.004, −0.002) <0.0001	−0.0017 (−0.005, 0.002) 0.32	−0.0021 (−0.005, 0.001) 0.20
**No**			
β (95% CI) *P*	−0.0013 (−0.004, 0.002) 0.44	−0.0024 (−0.004, −0.001) 0.0002	−0.0016 (−0.003, −0.0004) 0.01

Non-adjusted model (Model 1): None. Minimally adjusted model (Model 2): Age; sex; race. Fully adjusted model (Model 3): Higher education; marital status; daily sleepiness; moderate recreational activities; race; cigarette smoking; sex and age.

## Discussion

The present study showed a negative association between depression and daily consumption of caffeine lower than 90 mg. Meanwhile, the results varied when considering factors as race, smoking status, sex, education level and marital status.

A previous study indicated that caffeine intake has a significant inverse association with depression, concluding that the caffeine’s psychostimulant properties were able to protect against depressive symptoms (6). Some studies reiterated the complex association between caffeine intake and depression risk (11), and others have noticed the differences between males and females (12), so it is reasonable to include more confounders (variable Z) in future studies.

**FIGURE 4:**
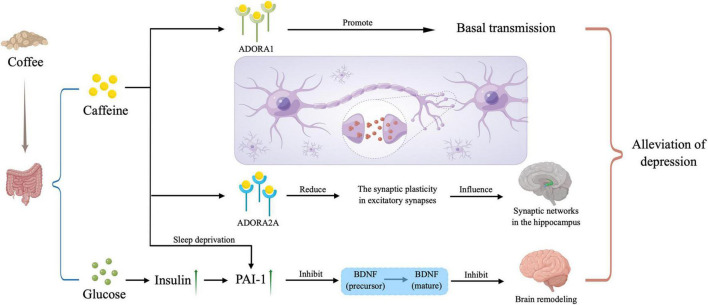
Potential mechanisms. Caffeine plays a role in the depression alleviation by combining with *ADORA1* and *ADORA2A* to influence our neural network in the brain, and inhibiting the mature of brain-derived neurotrophic factor (BDNF), and the latter way can also be triggered by large amounts of sugar taken in with caffeine when drinking coffee. PAI-1, plasminogen activator inhibitor; *ADORA1*, adenosine A1 receptor; *ADORA2A*, adenosine A2A receptor. The picture was drawn by Figdraw.

As for a mechanism, due to the similar structure, caffeine is able to compete with adenosine to combine with A1 receptors and A2A receptors (4, 13). When combining with A1 receptors, an increased basal transmission occurs (14). But the main mechanism is that after combining with A2A receptors, the synaptic plasticity decreased in excitatory synapses, which influences synaptic networks in the hippocampus to have the neuroprotective effects (15). And a cross-sectional population-based study shows that a single nucleotide polymorphism in A2A receptor gene has an association with depression (16), which also proves that A2A receptors play an important role in this negative association with depression. Besides, there exists two issues related to caffeine consumption. On the one hand, people often consume significant amounts of sugar with coffee, which causes the hyperinsulinemia and then stimulates plasminogen activator inhibitor (PAI)-1 production. So, the proteolytic cleavage of brain-derived neurotrophic factor (BDNF) precursor to mature BDNF is impaired by PAI-1 and brain remodeling in response to highly stressful situation or prolonged stress. On the other hand, sleep deprivation will be also influenced by the consumption of caffeine, resulting that cortisol levels and PAI-1 increase. Similarly, brain remodeling is prevented, and depression symptoms gets to relief. The mechanism above was shown in Figure 4.

In the present study, people who are female, younger, sleepier, have married but separated or haven’t married, have not received higher education, have less moderate recreational activities, tend to smoke, and have less caffeine intake are more likely to be depressive. A previous report found that people with lower education showed a higher ratio of depression (17). The difference might be due to the different academic demands (18). Smoking is also associated with depression. Depressive patients tend to smoke as a self-meditation because nicotine will combine with nicotinic-cholinergic receptors (nAChRs) and then stimulates dopamine release in the nucleus accumbens, relieving depression symptoms (19). While education status is not easy to change, doctors could offer the patients who smoke to relieve depression alternatives to regulate reward so that they will not depend on cigarette smoking, for example, caffeine (20), as patients with more caffeine intake had less PHQ-9 score when caffeine consumption is lower than 90 mg. In fact, the chronic exposure to caffeine can afford the neuroprotection against a lot of noxious stimulation to the brain besides depression, thus substituting nicotine as self-administration in many aspects (19). (Food and beverage which would provide 90 mg of caffeine was included in Table 5. A dose of 90 mg is provided by a large cup of instant coffee). The co-administration between caffeine and depression deserves further discussion, on the one hand, caffeine can enhance the release of dopamine induced by nicotine (21); on the other hand, the function of nAChRs was under the control of A2A receptor (caffeine is the antagonist of A2A receptor) (22).

**TABLE 5:** The consumption of food that contains 90 mg of caffeine (data provided by the USDA’s Food and Nutrient Database for Dietary Studies).

Food and beverages	Consumption
Not reconstituted instant coffee	2.87 g
Reconstituted instant coffee	346.15 g
Espresso	42.45 g
Brewed coffee	225.00 g
Mocha coffee	272.73 g
Cappuccino	250.00 g
Green tea	750.00 g
Black tea	450.00 g
Black chocolate	160.71 g
Regular cola	1000.00 g
Diet cola	750.00 g
Energy drink (Red Bull)	310.34 g

There are some limitations in the methods of the present study. First, this was a cross-sectional study based on a database, so only the association between caffeine intake and depression could be described while the casual connection cannot be accurately demonstrated. Also, only eight confounding factors were included, other potential confounding factors, such as trauma history, personal health history, income, and body mass index were not included. Only 3,263 samples were included in this study as only one cycle of the NHANES survey (2017–2018) was used and all samples with missing answers were excluded. The present study also excluded patients with too much caffeine intake (more than 500 mg) as too much caffeine can cause other health problems. Moreover, this study did not conduct clinical evaluations of depression, but merely used the data provided by NHANES using PHQ-9 depression scale to evaluate the depression level of patients, which would not be so accurate. Meditation wasn’t considered in the collection of caffeine intake, though caffeine is used in some medicine (23). Besides, the present study relied on a questionnaire rather than using the available biological measures to estimate the intake of coffee. In fact, although questionnaires have previously been shown to correlate with caffeine intake, the correlation is rather poor (circa 0.45) and it is observed that relations based on questionnaires are not always in line with data relying on biological estimates. Meanwhile, NHANES didn’t establish the amount of caffeine in drinking water according to different areas. The sugary content in the beverage wasn’t assessed, though previous study had found that hyperinsulinemia may contribute to the pathogenesis of depression (24). Another limitation of this study is that caffeine is only one of the xanthines acting as an antagonist of adenosine receptors, and other xanthines like theobromine (more abundant in cocoa/chocolate) is often more tightly related with alterations of brain function than caffeine (25) and theobromine is near equi-effective with caffeine to modulate the adenosine modulation system in the brain (26). Finally, most of the samples were non-Hispanic white.

In conclusion, the present study found that depression was negatively associated with caffeine intake (less than 90 mg). With the prevalence of depression reaching 22% worldwide, further research is needed to investigate the causal connection between caffeine and depression, and the different β value in different subgroups, to figure out whether and how caffeine could be applied to the treatment of depression. Also, in future studies the co-administration of glucose should be evaluated to elucidate its influence also on the affective state and depressive symptomatology.

## Data availability statement

The datasets presented in this study can be found in online repositories. The names of the repository/repositories and accession number(s) can be found in the article/Supplementary material.

## Ethics statement

The studies involving human participants were reviewed and approved by the National Center for Health Statistics (NCHS) Research Ethics Review Board (ERB). The patients/participants provided their written informed consent to participate in this study.

## Author contributions

JB: writing—original draft, formal analysis, writing—review and editing, data curation, and project administration. PL: writing—original draft, formal analysis, writing—review and editing, conceptualization, and software. YG: writing—original draft, formal analysis, writing—review and editing, and data curation. YZ, MS, and JH: writing—original draft, formal analysis, and writing—review and editing. All authors contributed to the article and approved the submitted version.
